# Optimization of the Organic Matter Content and Temperature in a Bioreactor to Enhance Carbon Monoxide Production During the Initial Phase of Food Waste Composting

**DOI:** 10.3390/molecules30132807

**Published:** 2025-06-30

**Authors:** Karolina Sobieraj

**Affiliations:** Department of Applied Bioeconomy, Wrocław University of Environmental and Life Sciences, 37a Chełmońskiego Street, 51-630 Wrocław, Poland; karolina.sobieraj@upwr.edu.pl

**Keywords:** carbon monoxide (CO) yield, organic matter content (OMC), temperature optimization, modeling, industrial scale, bulking agent, waste-to-chemicals, sustainable processes, carbon footprint reduction, green chemistry

## Abstract

Carbon monoxide (CO) is a key reactant in industries like chemicals, pharmaceuticals, and metallurgy, with a projected global market of $8.2 billion by 2032. A novel method of CO production is biowaste composting, but the impact of organic matter content (OMC) on CO yield remains unexplored. Since OMC affects composting costs, optimizing it is crucial for economic feasibility. This study aimed to identify the optimal OMC in bioreactors for CO production during food waste composting. A laboratory process was conducted in bioreactors with forced aeration. Food waste (FW) was mixed with gravelite (G) at ratios of 1:0, 1:1, and 1:2 (FW:G), corresponding to 95%, 40%, and 20% dry OMC. Bioreactors were incubated at 45 °C, 60 °C, and 70 °C with ~5% oxygen. The highest CO levels were at 70 °C for FW:G 1:2, with an average of 655 ppm and a maximum of 2000 ppm. Daily CO emissions were highest at 70 °C, reaching up to 1.25 mg. Therefore, the study demonstrated that even a low organic matter content allows for CO production during composting under thermophilic conditions (~70 °C) with limited oxygen. Industrial modeling estimated daily CO yield from 39.25 to 670.61 g, with a 7-day market value between USD 28.89 and USD 175.86. Further studies are needed for large-scale feasibility.

## 1. Introduction

Given that carbon monoxide (CO) poisoning is recognized as one of the leading global causes of morbidity and mortality, CO is predominantly associated with its detrimental effects on human health and life [1]. However, CO also possesses a lesser-known beneficial aspect, demonstrating remarkable versatility across various industries. The global CO market was valued at $5.6 billion in 2022 and is expected to reach $8.2 billion by 2032, reflecting a compound annual growth rate (CAGR) of 4.1% from 2023 to 2032 [2]. While itself a reactive gas, CO plays a crucial role as an intermediate in various CO_2_ conversion processes, including the synthesis of fuels and value-added chemicals. In the chemical industry, CO serves as a crucial reactant in the synthesis of numerous compounds, including acetic acid, formic acid, and precursors for polyurethane and polycarbonate plastics [3,4]. Furthermore, CO can be utilized in the production of methane, ethanol, and higher-order fuels [5]. Its utility has also been extensively studied and confirmed in fields such as medicine, the food industry, and the pharmaceutical sector [6]. CO, owing to its reducing capabilities, finds application in metallurgy [7]. Furthermore, preclinical studies have highlighted its potential therapeutic properties, particularly in mitigating cardiovascular dysfunction and inflammation in mammals [8,9,10]. Research has also shown that CO exposure can help preserve the color and aroma of fresh meat [11].

Currently, CO is produced using carbon-intensive processes such as the partial oxidation of hydrocarbons, steam reforming of natural gas, and coal gasification [5]. In the waste management sector, CO is obtained through the thermochemical process of biowaste gasification, which is associated with challenges such as high energy consumption and costly raw material preparation [6].

The most recently reported method of obtaining CO is the biowaste composting process. CO has been noted as a by-product of organic waste composting, including green waste (obtained CO concentration of 120 ppm [12], 120 μmol‧mol^−1^ [13]), animal waste (10 ppm [12], 10 μmol‧mol^−1^ [13]), plant waste (herbs residues, 160 ppm [12]), food waste (>2000 ppm [14]), the undersized fraction of municipal waste (112 μL‧L^−1^ [15], 222.7 g‧t^−1^‧h^−1^ [16]), green maintenance waste (0.04% of initial carbon content [17]), and a mixture of green waste with sewage sludge (>300 ppm [18,19]). Existing studies suggest that CO production is highest during the initial phase of the process. According to the literature, elevated CO concentrations were detected immediately after the formation of a composting pile from green waste [13] and within a few hours of starting the green waste composting experiment with grass [17,20]. When composting green waste, manure, and slurry, Hellebrand and Kalk [12] identified two peaks in CO production: the first occurring within a few hours of the process initiation (approx. 6–12 h), and the second emerging after 5–8 days. CO production typically declines sharply within the first days of the composting process. This trend was confirmed in our own study, where CO production decreased quickly and became negligible by day 14 under all tested conditions [21].

Studies conducted by Stegenta-Dąbrowska et al. [22] and Sobieraj et al. [6] have demonstrated that CO in compost can be produced through the biological activity of microorganisms. Although composting is an aerobic process, the underlying mechanisms of biotic CO formation are potentially anaerobic. Microbial CO production is primarily linked to the presence of the Wood–Ljungdahl (acetyl-CoA) pathway, which has been confirmed in anaerobic acetogens and methanogens [23]. Indeed, recent research on the functional capabilities of CO-producing microbial communities in biowaste and sewage sludge compost has revealed the presence of the Wood–Ljungdahl pathway in nearly all analyzed samples [6]. The bacterial production of CO was validated through analyses of bacterial strains isolated from compost, which, over 4 days of incubation, released CO at concentrations exceeding 1000 ppm under conditions of reduced oxygen availability [24]. Additionally, technical assessments of the biowaste composting process at the laboratory scale demonstrated that the highest CO concentrations in all tested thermal variants occurred under oxygen-deficient conditions [21].

While studies have examined the impact of aeration (thus oxygen concentration) on CO production, no research has yet explored the significance of organic matter content (OMC) in determining CO yield from the composting process. Although measurements of CO concentrations generated during the composting of various organic waste fractions varying in OMC have been conducted [12,13,14,15,16,17,21,22], direct comparison of these results remains challenging due to variations in composting techniques, process scale, duration, temperature, and aeration conditions. Since the organic load in the bioreactor (i.e., the required amount of organic waste with specific properties), along with process temperature and aeration, constitute the primary factors influencing composting costs, establishing the optimal OMC for CO generation during composting is essential for assessing the economic viability of this CO production method. Although laboratory-scale results do not allow for a comprehensive assessment of the economic efficiency of CO generation in composting and further research is required, the obtained data can be utilized as input for a model estimating potential CO yield on a technical scale.

Therefore, the study aimed to determine the optimal OMC in a bioreactor to enhance CO production during the initial phase of composting. Given the globally increasing amount of food waste [25], this fraction was composted to make the considered scenario more economically viable. Four bioreactor organic matter loadings were analyzed, ranging from 0% dry OMC to 20%, 40%, and 95% dry OMC. Considering reports indicating the most intense CO production occurs in the first week of the process under quasi-anaerobic conditions, laboratory-scale composting was conducted for seven days with a reduced oxygen supply. To determine the optimal temperature for CO release, the bioreactors were incubated under mesophilic (45 °C and 60 °C) and thermophilic (70 °C) conditions. The laboratory-scale results were then used to model the potential CO yield in a full-scale composting facility.

## 2. Results

### 2.1. Biowaste and Compost Characterization

The dry matter content in substrates varied from 20.9% in the 1:0 variant to 57.0% in the 1:2 mix, with OMC decreasing as gravelite content increased, reaching 95.3% d.m. for the highest food waste proportion and 21.5% for the 1:2 mix (LOI) (Table 1). Higher gravelite content reduced AT_4_, peaking at 27.5 mg O_2_∙g d.m.^−1^ for food waste alone and dropping to 8.5 mg O_2_∙g d.m.^−1^ for the 1:2 sample. Elemental analysis showed carbon (C) as the dominant element, ranging from 44.7% (1:0) to 3.2% (1:2), followed by hydrogen (H), nitrogen (N), and sulfur (S). Pure gravelite remained unchanged before and after processing (100% d.m., LOI = 0, AT_4_ = 0).

After composting, similar trends were observed: increased dry matter, decreased LOI, and AT_4_ reduction with lower food waste content. For the 1:0 variant, dry matter reached 21–23% after seven days, while LOI remained stable (~95% d.m.). AT_4_ peaked at 23.1 mg O_2_∙g d.m.^−1^ at 45 °C, decreasing to ~17 mg O_2_∙g d.m.^−1^ at higher temperatures. Dry matter for 1:1 and 1:2 remained consistent across temperatures (46.1–47.6% and 54.3–56.0%, respectively), with LOI increasing with temperature, peaking at 56.0% d.m. (1:1, 70 °C). Most 1:1 and 1:2 composts were non-reactive (AT_4_ < 10 mg O_2_∙g d.m.^−1^), except for 1:1 at 60 °C (14.6 mg O_2_∙g d.m.^−1^).

Elemental composition of 1:0 composts showed minor changes, with C content increasing slightly at 60 °C (44.7% to 47.1%), while thermophilic conditions slightly reduced all elements. In 1:1 and 1:2 variants, C, H, N, and S increased after seven days, with 1:1 composts containing 23.3–27.8% C, 3.1–4.0% H, and lower N and S levels. The 1:2 variant had <20% C, <3% H, and <0.8% S. Gravelite composted alone at 45 °C showed low elemental content, with H predominance (2.7%), while C and N were minimal at higher temperatures, and H and S were undetectable.

### 2.2. CO, O_2_ and CO_2_ Production During Laboratory-Scale Composting

**Figure 1:**
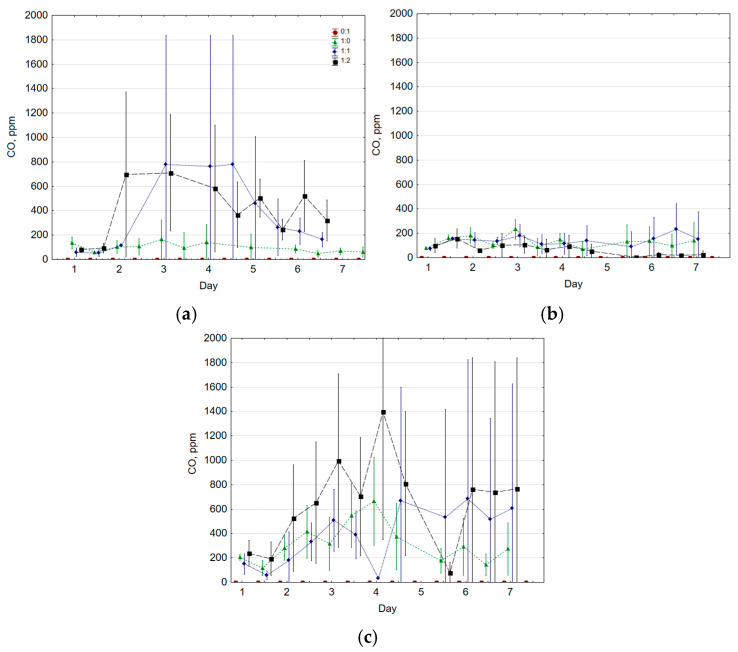
CO concentration during composting at (**a**) 45 °C; (**b**) 60 °C; (**c**) 70 °C (average ± standard deviation).

Research indicated that the highest average CO concentrations over the entire seven-day composting period were obtained in the variant with the highest temperature (70 °C), regardless of the FW:G ratio used (Figure 1). The peak CO production at 45 °C and 70 °C was recorded between the third and fifth day of reactor incubation (approx. 800 and 1400 ppm on average for 45 °C and 70 °C, respectively). In comparison, for 60 °C, CO generation was lower and more stabilized for seven days of the process. Interestingly, this thermal variant, considered as with the optimal composting temperature, had the lowest CO yield (on average 131, 140, and 68 ppm for the 1:0, 1:1, and 1:2 ratios, respectively). Importantly, gravelite incubated alone (FW:G of 0:1) did not produce CO in any temperature variant.

The highest average CO concentrations were obtained for a FW:G ratio of 1:2 in the 45 °C and 70 °C variants (412 and 655 ppm, respectively). In the former, the CO peak was indeed produced at a FW:G ratio of 1:1, but the gas production was more stable throughout the process for the 1:2 variant and reached a higher average value (366 and 412 ppm for 1:1 and 1:2, respectively). Detailed trendline analysis and additional plots, including polynomial equations fitted to the average CO concentrations for each experimental variant along with corresponding R^2^ values, are provided in the Supplementary Material S4.

**Figure 2:**
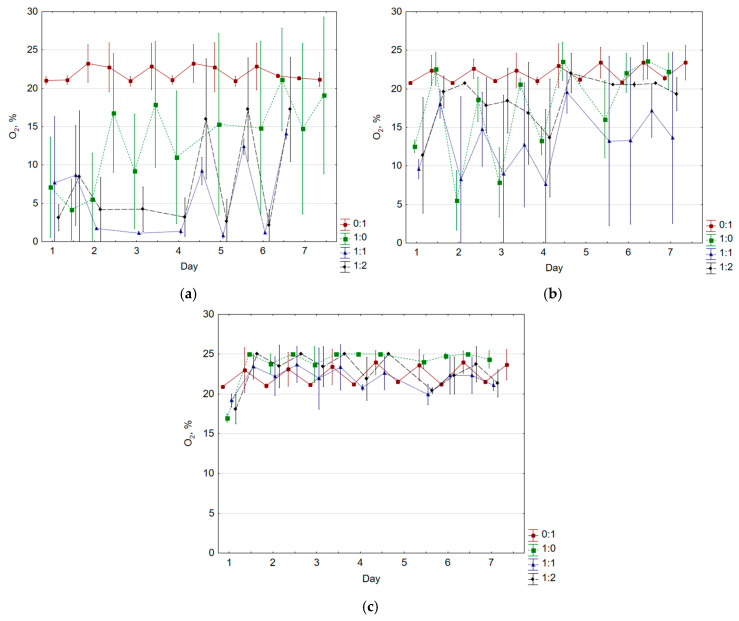
Oxygen concentration during composting at (**a**) 45 °C; (**b**) 60 °C; (**c**) 70 °C (average ± standard deviation).

The oxygenation level in bioreactors varied with incubation temperature (Figure 2). Higher temperatures led to greater oxidation in the headspace. At 45 °C, hypoxic conditions prevailed in lower OMC variants (>10%, averaging 5.7% and 9.6% for 1:1 and 1:2, respectively) for the first 4.5 days, while 1:0 started at 8.8% O_2_ but reached 25.0% by day 7. Oxygenation was slightly higher at 60 °C, with occasional O_2_ levels below 10% in 1:0 and 1:1 but generally high otherwise. Under thermophilic conditions, O_2_ levels remained high (>16%). Oxidation in gravelite bioreactors stayed at 20–25% at all temperatures.

CO and O_2_ concentrations were negatively correlated at 45 °C and 60 °C (Table S1, Supplementary Material S5). At 60 °C, the correlation weakened with lower OMC (−0.34 to −0.63 for FW:G of 1:0 to 1:2). At 45 °C, food waste alone (FW:G 1:0) showed a stronger negative correlation (r = −0.65). At 70 °C, O_2_ concentration stimulated CO production in FW:G 1:1 and 1:2 (r = 0.33 and 0.42).

Under mesophilic conditions, O_2_ and CO_2_ were inversely correlated (−0.76 to −0.90 at 45 °C, −0.73 to −0.84 at 60 °C, Table S1). At 45 °C, CO_2_ was highest in lower OMC variants, peaking at 39%. At 60 °C, lower CO_2_ levels were observed, with maxima of 12.2% (1:0) and 15.9% (1:1). Under thermophilic conditions, CO_2_ remained low (<10%), with a peak of 13% on day 4 in the lowest OMC variant.

CO and CO_2_ were positively correlated across most temperatures and OMC variants (Table S1). Higher temperatures strengthened this correlation, with the highest value at 70 °C for FW:G 1:2 (r = 0.96).

### 2.3. CO Mass in Bioreactors

**Figure 3:**
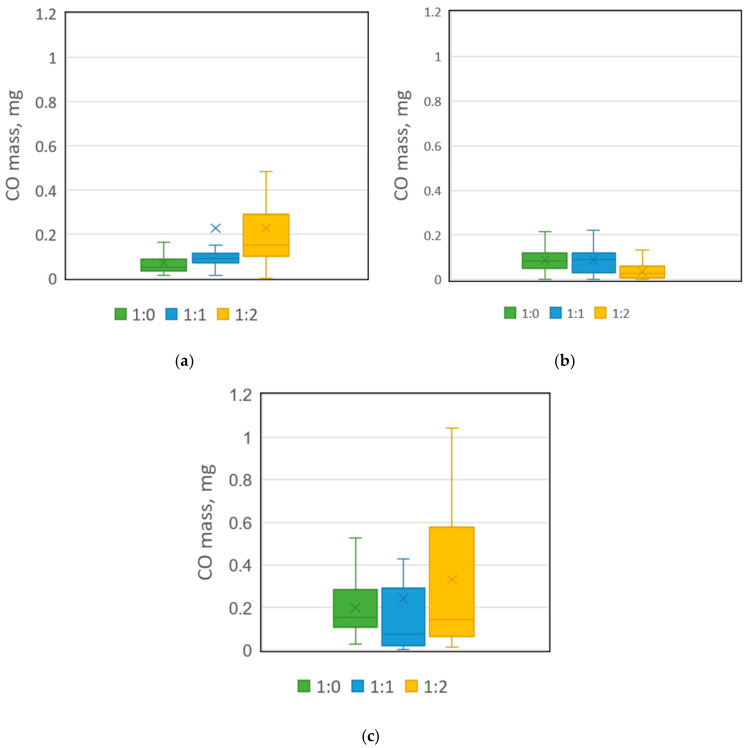
Average CO mass emitted during 7 days of composting process in bioreactors incubated at (**a**) 45 °C, (**b**) 60 °C, (**c**) 70 °C.

The daily emitted CO mass in bioreactors varied from 0.01 to 1.29 mg, depending on applied thermal conditions and the FW:G ratio (Supplementary Material S1). In general, the results showed that the highest CO mass among the OMC variants was obtained at 70 °C (Figure 3). The average CO mass at this temperature was 0.20, 0.24, and 0.34 mg for FW:G ratios of 1:0, 1:1, and 1:2, respectively. However, the maximum daily values for these OMC variants at 70 °C were much higher, reaching 0.68, 1.25, and 1.04 mg CO, respectively (Figure 3, Supplementary Material S1). Higher maximum daily values were recorded only in the FW:G of 1:1 variant at 45 °C, where the CO mass reached 1.29 mg (Supplementary Material S1). The lowest CO amount was obtained at 60 °C, where the average mass did not exceed 0.09 mg, and the highest recorded daily CO production was 0.27 mg.

Expressing the efficiency of CO yield from substrates by the daily CO mass index, the highest CO production variants recorded maximum values of 16.5 mg CO∙kg FW^−1^ (10.1 mg CO∙(kg FW + G)^−1^) for an FW:G ratio of 1:1 at 45 °C, 15.2 mg CO∙kg FW^−1^ (6.3 mg CO∙(kg FW + G)^−1^) for the 1:2 variant at the same temperature, and 17.6 mg CO∙kg FW^−1^ (7.5 mg CO∙(kg FW + G)^−1^) for the 1:2 mix under thermophilic conditions (Supplementary Material S1).

### 2.4. CO Yield in Technical-Scale Composting Plant

**Figure 4:**
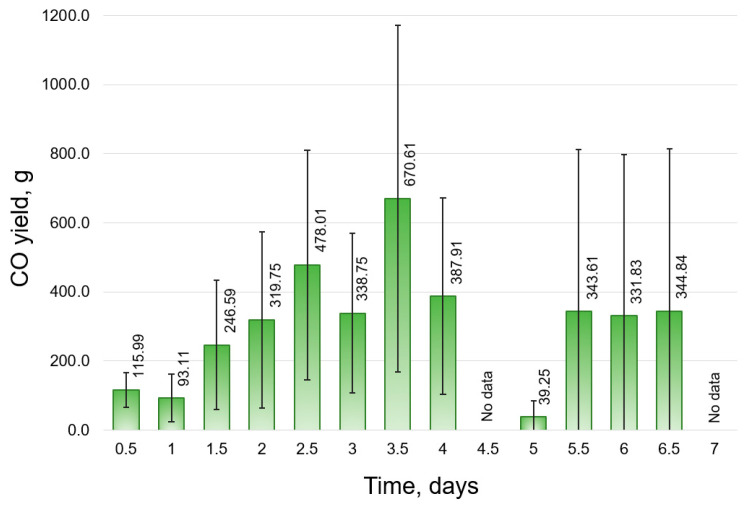
Daily CO yield in bioreactor modeled for technical-scale composting plant (average ± standard deviation); no values available for days 4.5 and 7 due to the lack of input data for the model.

Modeling has demonstrated that in the industrial composting facility operating the closed bioreactor under thermophilic conditions, the daily CO yield during 7 days of a process can range, on average, from 39.25 to 670.61 g (Figure 4). The CO production exhibited two peaks: the first occurring between days 2.5 and 4 of the composting process, during which the highest CO mass levels exceeding 1 kg were recorded (1032 g CO at the midpoint of day 3, as presented in Supplementary Material S2), and the second characterized by a relatively stable daily emission rate between days 5.5 and 6.5 (with an average daily value of approx. 340 g CO and peak yield reaching ~800 g CO).

The daily efficiency of CO yield in the industrial composting facility was modeled within a range of 0.2 to 16.4 mg CO∙kg FW^−1^ (7.5 mg CO∙(kg FW + G)^−1^), with an average value of 4.9 mg CO∙kg FW^−1^ (2.3 mg CO∙(kg FW + G)^−1^, Supplementary Material S2).

## 3. Discussion

Since the literature contains very few studies on the mechanisms of CO production during composting, it is necessary to seek analogous ecosystems to which the obtained results and observations can be compared. One such ecosystem is soil, which is widely reported as both a source and a sink of CO through biotic and abiotic processes [4]. Researchers acknowledge that photodegradation of organic compounds significantly contributes to CO release from soil [26]. However, this process, involving visible and UV radiation breaking carboxyl bonds and releasing CO [27], is not applicable to the presented study. Here, food waste in the bioreactors was incubated in closed climate chambers without exposure to sunlight, thereby preventing the influence of solar radiative energy on the substrates and their organic components.

The study demonstrated that the concentration of CO produced during food waste composting was inversely correlated with OMC, with the highest average CO production occurring at an FW:G ratio of 1:2 at 45 °C and 70 °C. This trend contrasts with the findings reported in the literature, which indicate that abiotic CO production at relatively low temperatures (<100 °C) occurs predominantly in carbon-rich ecosystems, as observed in studies of peatlands and arid soils [26,28]. Research conducted as early as 1996 by Conrad [29] indicated that in soil, CO is produced through the thermal decomposition of humic acids and other organic material, with CO emissions being strongly dependent on high OMC and temperature. Other reports support this, suggesting that CO is a by-product of the thermal degradation of carbon from senescent plant material [30]. This physicochemical process has also been identified as a source of CO release during composting [12,17]. Furthermore, Hellebrand and Kalk [12] hypothesized that in the initial phase of the composting process, thermal degradation is the dominant cause of CO emissions. They supported this claim by demonstrating an increase in CO production with rising temperature, which was positively correlated with O_2_ availability. The same results (i.e., a positive correlation of CO concentration with O_2_ level at 70 °C) were observed in the present study, suggesting that thermochemical CO production likely occurred under thermophilic conditions.

The higher CO production with lower OMC observed in this study may indicate the biological production of CO by microorganisms in the compost, particularly at 45 °C, where the highest daily CO concentration maxima were recorded. The activity of the microbial community producing CO under these conditions could have been influenced by the high proportion of gravelite in the bioreactors (FW:G of 1:2). While gravelite did not produce CO, it may have facilitated its formation. Mineral additives have been reported to indirectly alter microbial activity through their impact on temperature, oxidation, and moisture of the composted waste [31]. Gravelite present in the bioreactors with the highest CO production may have provided not only structural support to create inter-particle voids but also a surface for microbial growth [32]. The results obtained in this study are consistent with the findings of Gea et al. [33] and Eftoda and McCartney [34], who demonstrated that the optimal free air space in compost was achieved for a mix of substrates with bulking agents at ratios of 1:1 and 1:2.5, respectively. The beneficial increase in porosity of the compost matrix has also been confirmed for inorganic bulking agents such as rock phosphate and pumice [35].

The hypothesis regarding the biological basis of CO production at 45 °C in this study may also be supported by the observed correlation between CO and O_2_ concentrations. Under mesophilic conditions, an inverse correlation between the levels of these gases was noted. This trend has been observed in previous studies and was associated with the presence of anaerobic mesophiles in the composting mass, such as *Alkalibaculum bacchi*, *Butyribacterium methylotrophicum*, *Acetobacterium woodii*, and *Rhodospirillum rubrum* [21]. These strains have been reported to possess the ability to produce the enzyme CO dehydrogenase (CODH), which facilitates both the consumption and production of CO through the bidirectional reaction: CO + H_2_O ⇌ CO_2_ + H_2_ [23]. Some of these strains are facultative anaerobes and have previously been isolated from analogous to compost ecosystems such as livestock-impacted soil [36,37].

Regardless of the nature of CO production during composting (biotic vs. abiotic), the trend observed in this study—where higher temperatures resulted in increased CO yield—was consistent with the findings of other researchers [21,22,28]. From a practical perspective, optimizing bioreactor performance to enhance CO production requires precise regulation of both temperature and OMC during the composting process. According to the results, to intensify CO production, the process should be conducted under thermophilic conditions (~70 °C) with a limited oxygen supply (oxygenation at approx. 5%). Researchers have previously acknowledged the proposed elevated temperature as beneficial for composting. Beyond its well-documented role in pathogen inactivation (waste hygienization) [38], it has also been associated with accelerated organic matter decomposition during thermophilic composting [39]. Moreover, studies dating back to the 1960s identified 70 °C as the optimal temperature for composting, based on oxygen uptake rate (OUR) measurements [40].

Given that the highest CO generation occurs between the second and fourth day of the process, followed by a secondary peak in CO concentration on the sixth and seventh days, for CO production, it is recommended to focus on the initial composting phase (the whole 1st week). Effective process control aimed at maximizing CO yield also requires careful optimization of OMC within the bioreactor. The findings indicate that the most favorable FW:G ratio is 1:2, suggesting that even a relatively small amount of organic matter suffices to facilitate CO production during composting, yielding superior outcomes compared to processes with higher OMC levels. Gravelite, which has been employed as a bulking agent to reduce OMC in the bioreactor, may be substituted with other widely available, slow-decomposing materials such as wood. Lignocellulosic waste, commonly utilized in composting, offers several advantages, including the absence of odor emissions during decomposition, low concentrations of potentially toxic elements and other pollutants, and easy separation from other materials [41].

The maximum daily CO mass values obtained in bioreactors during this study, expressed per unit mass of organic substrate, ranged between 15.2 and 17.6 mg CO∙kg FW^−1^, exceeding those previously reported in the literature. In particular, this study achieved a daily CO yield > 15 mg CO∙kg FW^−1^ at 45 °C, whereas Hellebrand and Kalk [12] under comparable conditions (laboratory scale, 50 °C) observed CO production at a level of 11.0 mg CO∙kg substrate^−1^. Additionally, at 70 °C, the highest daily CO production recorded here was 17.6 mg CO∙kg FW^−1^, while the same researchers reported a value of 7.9 mg CO∙kg substrate^−1^ at 65 °C. A possible factor contributing to these differences is the duration of the process. This study focused on the initial phase of composting lasting 7 days (168 h), while Hellebrand and Kalk [12] presented results for a composting period of 480 h. Given previous reports indicating a decline in CO emissions from composting after 1–2 weeks [16], cumulative CO production for 20 days may have been lowered due to prolonged periods of reduced gas release.

Analyzing analogous results for the technical scale, the values obtained by other researchers vary notably. While the modeling conducted in this study indicated the maximum daily efficiency of CO yield in the industrial composting facility to be 16.4 mg CO∙kg FW^−1^, Hellebrand [17] reported CO emissions of 0.11 mg CO∙kg of substrate^−1^ (0.40 mg CO∙kg of carbon content^−1^) during the composting of green waste from land maintenance. Similarly, Andersen et al. [42], who investigated gas emissions from a garden waste pile, observed CO emissions of 0.15 mg∙kg of wet waste mass^−1^. However, it should be emphasized that these comparisons, both at the laboratory scale and those derived from modeling for the industrial scale, should be treated as rough estimates. This is due to the use of different waste materials in the composting process (which affects the OMC), differences in process duration, and composting methods, which, as discussed in the introduction, prevent a precise comparison of the obtained results. Additionally, the process conducted by Hellebrand [17] was carried out in open piles rather than enclosed reactors. In this case, atmospheric conditions (e.g., wind effects) could have influenced the determination of CO emission levels. CO, being slightly lighter than air [43], could rapidly ascend, thereby distorting the results. On the other hand, it is also worth highlighting that in Andersen et al.’s [42] study, advanced analytical methods, such as the dynamic plume method with a mobile FTIR system, were employed to determine CO emission levels. These methods allowed for highly accurate quantification of CO production.

Considering the assumptions adopted in the model, the maximum daily mass yield of CO during the initial phase of composting on the industrial scale exceeded 1 kg CO. This quantity can be expressed in terms of the market value of CO. A market analysis conducted in 2021 indicated an upward trend in CO prices across North America, Europe, and the Asia–Pacific region. Based on a 2021 price of USD 28,708 per metric ton (MT) [44], the maximum daily CO production estimated in the modeled composting process corresponded to a market value of USD 29.63. Assuming daily extraction of CO from the bioreactor headspace, the total value of the CO produced over 7 days ranged from USD 28.89 to USD 175.86 for a CO mass of 1.01 to 6.13 kg. It is noteworthy that this value was modeled under standard composting conditions, with an optimized OMC and temperature to maximize CO production. Conducting the composting process in dedicated bioreactors, with all parameters adjusted to enhance CO release, could further increase this value.

Although the estimated market value of CO from industrial-scale composting in the developed model is relatively low, it is important to emphasize that quantity may play a crucial role in the overall profitability of the project. Data from 2022 indicate that composting is the predominant method for processing biowaste in Europe. Currently, 3800 operating composting plants provide extensive technical infrastructure that could be adapted for CO production, and projections suggest that this number will increase to 7700 by 2035 [45]. These favorable trends could significantly contribute to the development of the bioeconomy by integrating the biowaste composting process with simultaneous CO production.

## 4. Materials and Methods

### 4.1. Food Waste Composition

**Figure 5:**
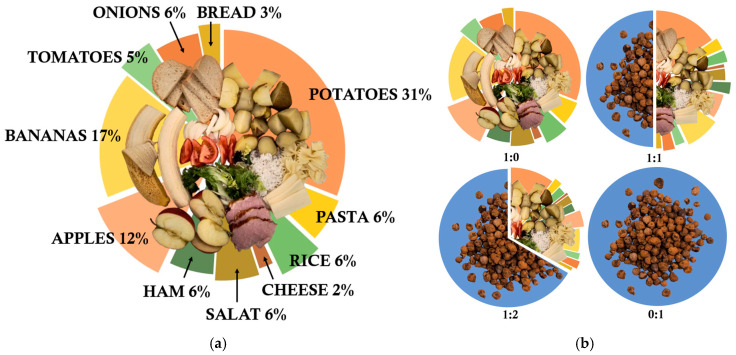
Bioreactor input during laboratory-scale composting: (**a**) food waste composition; (**b**) experimental variants with different food waste to gravelite ratio (FW:G, *v*/*v*): 1:0, 1:1, 1:2, 0:1.

A model food waste mixture (FW), prepared based on [46], was composted (Figure 5a). The food waste input consisted of cooked potatoes, rice, and pasta, along with fresh onions, apples, bananas, tomatoes, lettuce, bread, cheese, and ham, all cut into pieces of minimum 2 cm in size. To regulate the organic matter content within the bioreactor, the FW was combined with a mineral bulking agent, gravelite (G), with ratios: 1:0, 1:1, and 1:2 (FW:G, *v*/*v*), corresponding to 95, 40, and 20% dry OMC (Figure 5b). As a control, gravelite alone was also composted (ratio 0:1, 0% OMC). The particle size of gravelite used in this study was ~10 mm. The total mass of the feedstock in the bioreactor depended on the applied FW:G ratio and ranged from approx. 130 g to 170 g.

### 4.2. Food Waste Composting on a Laboratory Scale

**Figure 6:**
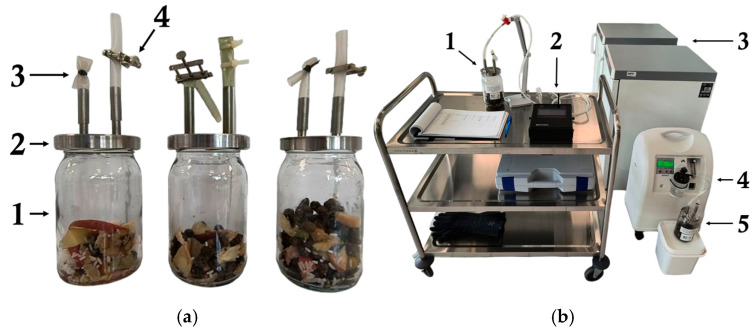
Experimental set-up of food waste composting on a laboratory scale: (**a**) bioreactor module: 1—900 mL glass bioreactors, 2—metal cap with two connectors, 3—sealed silicone tube, 4—silicon tube with Hoffmann clamp; (**b**) work unit structure: 1—gas concentration measurement unit, 2—portable gas analyzer, 3—thermostatic cabinets, 4—oxygen concentrator, 5—aeration unit.

The composting process lasted 7 days and was conducted in triplicate in 900 mL bioreactors with forced aeration (Figure 6). A 7-day duration was selected based on the literature and preliminary observations indicating that CO production typically declines sharply within the initial days of composting and becomes negligible by day 14 [19,21]. The bioreactors were placed in thermostatic cabinets (ST3, POL-EKO, Wodzisław Śląski, Poland) maintained at temperatures of 45 °C, 60 °C, and 70 °C. Each bioreactor had a metal cap featuring two connectors and silicone tubing. One connector remained sealed, while the other was opened and closed using a Hoffmann clamp to allow connection to a portable gas analyzer (DP-28, Nanosens, Wysogotowo, Poland). Gas concentration measurements for CO (ppm), CO_2_ (%), and O_2_ (%) were taken twice daily at 9:00 AM and 4:00 PM, each time for approx. 2 min until the indicated values stabilized. After each gas concentration measurement, the analyzer was disconnected for a short pause and used again after returning to ambient levels (CO~0 ppm, CO2 ~0%, O_2_~20.2%). Aeration was provided daily for 1.5 min using an oxygen concentrator (OxyFlow-10, GESS, Lubuskie, Poland) at a flow rate of 10 dm^3^∙min^−1^ (corresponding to weekly oxygenation of 5%), immediately after the first gas concentration measurements.

### 4.3. Substrates and Composts Characterization

The substrates and the material collected after 7 days (hereinafter referred to as ‘compost’)—representing the entire contents of the bioreactor, amounting to 130–170 g—were used for material characterization analyses. These included dry matter content and loss on ignition (LOI) according to the relevant standards [47,48]. The ultimate elemental composition analysis (C, H, N, S) was performed using a Perkin Elmer 2400 Series analyzer (Waltham, MA, USA) following the PN-EN ISO 16948:2015-07 standard [49]. The respiratory activity AT_4_ was determined using the OxiTop Control measuring system, Weilheim, Germany, in accordance with Binner et al. [50]. The bulk density of food waste and gravelite was analyzed following the standard [51]. All analyses were performed in three replicates.

### 4.4. Analytical Procedures

#### 4.4.1. Calculation of Daily Emitted CO Mass

CO concentration in the headspace of bioreactors in ppm was converted to normalized mass according to the following equation [52]:

(1) $$C_{gas}=C_{ppm}\cdot \frac{MW\cdot P}{R\cdot T_{r}}$$

where

C_gas_—CO concentration, mg∙m^−3^;

C_ppm_—CO concentration in parts per million, ppmv;

MW—molecular weight of CO, MW = 28 g∙mol^−1^;

P—atmospheric pressure, P = 101.32 kPa;

R—ideal gas law constant, R = 8.314 m^3^∙Pa∙K^−1^∙mol^−1^;

T_r_—the temperature in the bioreactor, K.

The headspace volume in the bioreactor (defined as the volume above the substrates) was calculated using the determined bulk density of food waste and gravelite according to the following equation:

(2) $$V_{headspace}=V_{bioreactor}-\frac{m_{FW}}{\rho_{FW}}-\frac{m_{G}}{\rho_{G}}$$

where

V_headspace_—headspace volume in the bioreactor, m^3^;

V_bioreactor_—the volume of the bioreactor, V_bioreactor_ = 0.0009 m^3^;

m_FW_—mass of food waste in the bioreactor, kg;

ρ_FW_—bulk density of food waste, ρ_FW_ = 654.1 kg∙m^−3^;

m_G_—mass of gravelite in the bioreactor, kg;

ρ_G_—bulk density of gravelite, ρ_G_ = 278.4 kg∙m^−3^.

The daily mass of emitted CO in the bioreactor was determined using the following formula:

(3) $$m_{CO}=C_{gas}\cdot V_{headspace}$$

where

m_CO_—the mass of daily emitted CO in the bioreactor, mg.

Calculations made for all FW:G variants at 45 °C, 60 °C, and 70 °C are presented in an Excel spreadsheet in Supplementary Material S1.

#### 4.4.2. Model of CO Yield in a Composting Plant

For modeling of CO yield in a composting plant, it was assumed that food waste was composted on a technical scale in an enclosed bioreactor commonly used in large waste management facilities. The process was conducted in a horizontal static reactor with a rectangular cross-section tunnel (composting unit) with forced aeration from the bottom [52]. Based on Global Composting Solutions Ltd., Mason and Milke [53,54], the working volume of the bioreactor was established as 500 m^3^ with dimensions of 5 × 5 × 20 m (width × height × length). In the bioreactor, the waste-to-headspace volume ratio of 4:1 was set. The organic substrates’ bulk density was set to 472 kg∙m^−3^ according to Hemidat et al. [55] to simulate real composting conditions, including the addition of structural material to food waste. The modeling focused on the initial phase of composting (the first 7 days), conducted under thermophilic conditions (70 °C) with a ratio of organic to inorganic substrate volume of 1:2 (OMC equal to 20%). All model calculations are presented in an Excel spreadsheet in Supplementary Material S2 (sheets: ‘Model inputs’ and ‘Model’).

### 4.5. Statistical Analyses

Data were analyzed using Statistica 13 StatSoft Inc. (San Ramon, CA, USA), TIBCO Software Inc. (San Ramon, CA, USA), including estimating the measurements’ mean and standard deviation, and correlation analysis (CO concentration vs. OMC, CO concentration vs. CO_2_, O_2_ concentrations). Polynomial trendline equations for CO concentration average values have been fitted with the corresponding R^2^ using Excel, Microsoft Office 16 (Redmond, WA, USA).

## 5. Conclusions

The presented research introduces a novel and sustainable method for carbon monoxide (CO) production via controlled composting of food waste, an approach that not only addresses organic waste management challenges but also contributes to climate change mitigation. By enabling renewable CO production from biowaste, the study proposes a pathway where CO serves as a bridge molecule, transforming biogenic carbon into usable forms, thereby offsetting the need for fossil-based CO and reducing net CO_2_ emissions.

The conducted study not only presented the first fundamental engineering approach to enhancing CO production during food waste composting but also quantified CO yields achieved at the laboratory scale and the potential CO amount that could be obtained in industrial composting facilities. The analyses demonstrated that even a small amount of organic matter in the bioreactor (OMC of approx. 20%) enables CO production during composting, yielding better results compared to higher OMC levels. According to the findings, to intensify CO production, the process should be conducted under thermophilic conditions (~70 °C) with a limited O_2_ supply (oxygenation at 5%). To fully characterize the influence of process parameters on CO yield during composting, future studies should complement the present results with analyses of CO production at varying substrate moisture levels.

The study has certain limitations. During the laboratory-scale composting of food waste, pure O_2_ (93–96%) was supplied to the process. In real-world conditions, such aeration is not employed. Therefore, any economic feasibility assessment of CO production from the composting process would need to account for the use of air instead of pure O_2_. Furthermore, in composting, food waste is not used as the sole substrate, as was presented in this study. Instead, it is mixed with other fractions of organic waste to obtain substrates with appropriate properties. However, the research presented here identifies the optimal OMC in the bioreactor for CO production. This knowledge can be applied to other substrates in the composting process, as long as the OMC remains consistent with the optimal value determined in this study.

Since the potential CO yield in an industrial composting facility was modeled in this study, it is recommended to scale up the research and conduct quantitative analyses of CO production from an actual compost pile at a technical scale. Such pilot studies would allow for a comprehensive economic assessment of CO yield from composting.

## Figures and Tables

**Table 1:** Biowaste and compost characterization (average ± standard deviation); <DL—below detection limit.

Variant	FW:G Ratio	Dry Matter (d.m.), %	LOI, % d.m.	AT_4_, mg O_2∙_g d.m.^−1^	C, %	H, %	N, %	S, %
Substrates	0:1	100 ± 0.0	0.0 ± 0.1	0.0 ± 0.0	0.3 ± 0.0	<DL	0.3 ± 0.2	<DL
	1:0	20.9 ± 0.0	95.3 ± 0.3	27.5 ± 0.3	44.7 ± 1.9	7.0 ± 0.7	2.4 ± 0.5	1.5 ± 0.0
	1:1	45.1 ± 0.0	39.1 ± 2.3	12.2 ± 0.8	8.6 ± 0.2	0.8 ± 0.2	0.6 ± 0.1	0.5 ± 0.2
	1:2	57.0 ± 0.0	21.5 ± 2.1	8.5 ± 1.2	3.2 ± 0.6	0.3 ± 0.3	0.4 ± 0.2	0.5 ± 0.0
45 °C	0:1	100 ± 0.0	0.0 ± 0.1	0.0 ± 0.0	0.4 ± 0.2	2.7 ± 2.1	1.3 ± 0.9	0.8 ± 0.5
	1:0	23.0 ± 0.0	95.0 ± 1.0	23.1 ± 1.2	44.0 ± 0.7	6.3 ± 0.4	2.2 ± 0.1	1.4 ± 0.1
	1:1	46.1 ± 0.1	28.4 ± 3.2	7.5 ± 0.2	27.8 ± 8.9	4.0 ± 1.3	2.4 ± 0.9	1.1 ± 0.2
	1:2	54.3 ± 0.1	18.1 ± 7.5	6.4 ± 0.5	19.6 ± 13.2	2.7 ± 2.1	1.3 ± 0.9	0.8 ± 0.5
60 °C	0:1	100 ± 0.0	0.0 ± 0.1	0.0 ± 0.0	0.4 ± 0.0	<DL	0.3 ± 0.1	<DL
	1:0	22.7 ± 3.4	95.2 ± 0.7	17.8 ± 2.3	47.1 ± 3.2	7.1 ± 0.4	2.8 ± 0.9	1.7 ± 0.2
	1:1	36.1 ± 3.5	40.1 ± 4.3	14.6 ± 2.4	23.3 ± 19.3	3.1 ± 2.6	0.8 ± 0.6	0.9 ± 0.7
	1:2	55.5 ± 8.8	17.0 ± 6.8	5.9 ± 1.1	13.4 ± 2.7	1.8 ± 0.5	0.5 ± 0.1	0.5 ± 0.1
70 °C	0:1	100 ± 0.0	0.0 ± 0.1	0.0 ± 0.0	0.5 ± 0.0	<DL	0.3 ± 0.1	<DL
	1:0	21.1 ± 2.6	96.4 ± 0.6	17.3 ± 1.9	44.4 ± 1.2	6.2 ± 0.3	1.8 ± 0.6	1.4 ± 0.2
	1:1	47.6 ± 6.9	56.0 ± 15.5	10.0 ± 2.5	26.5 ± 3.0	3.7 ± 0.4	1.6 ± 0.3	1.0 ± 0.1
	1:2	56.0 ± 7.0	28.3 ± 8.8	6.3 ± 0.8	15.4 ± 11.4	2.0 ± 1.6	0.8 ± 0.4	0.5 ± 0.3

## Data Availability

Raw data supporting reported results can be found in the repository: DOI:10.57755/s1b5-cm63.

## Supplementary Materials

The following supporting information can be downloaded at https://www.mdpi.com/article/10.3390/molecules30132807/s1: Supplementary Material S1—an Excel spreadsheet with 9 sheets showing calculations of daily emitted CO mass made for all FW:G variants at 45 °C, 60 °C, and 70 °C; Supplementary Material S2—an Excel spreadsheet with 2 sheets showing model inputs and model calculations for CO yield from industrial-scale composting; Supplementary Material S3—a Word file with Figure S1 showing CO_2_ concentrations in the bioreactors headspace during composting at all temperature and FW:G ratios variants; Supplementary Material S4—an Excel spreadsheet with polynomial equations fitted to the average CO concentrations for each experimental variant along with corresponding R^2^ values; Supplementary Material S5—an Excel spreadsheet with one sheet showing Table S1 with correlation analysis results between CO and CO_2_, O_2_ concentrations.
