# Supplementary material for: Optimization of the Organic Matter Content and Temperature in a Bioreactor to Enhance Carbon Monoxide Production During the Initial Phase of Food Waste Composting
Source: Molecules. 2025 Jun 30;30(13):2807. doi: 10.3390/molecules30132807 (PMC12251238; doi:10.3390/molecules30132807)
Supplement: Supplementary file 1 [file molecules-30-02807-s001.zip › Supplementary Material S3.pdf]

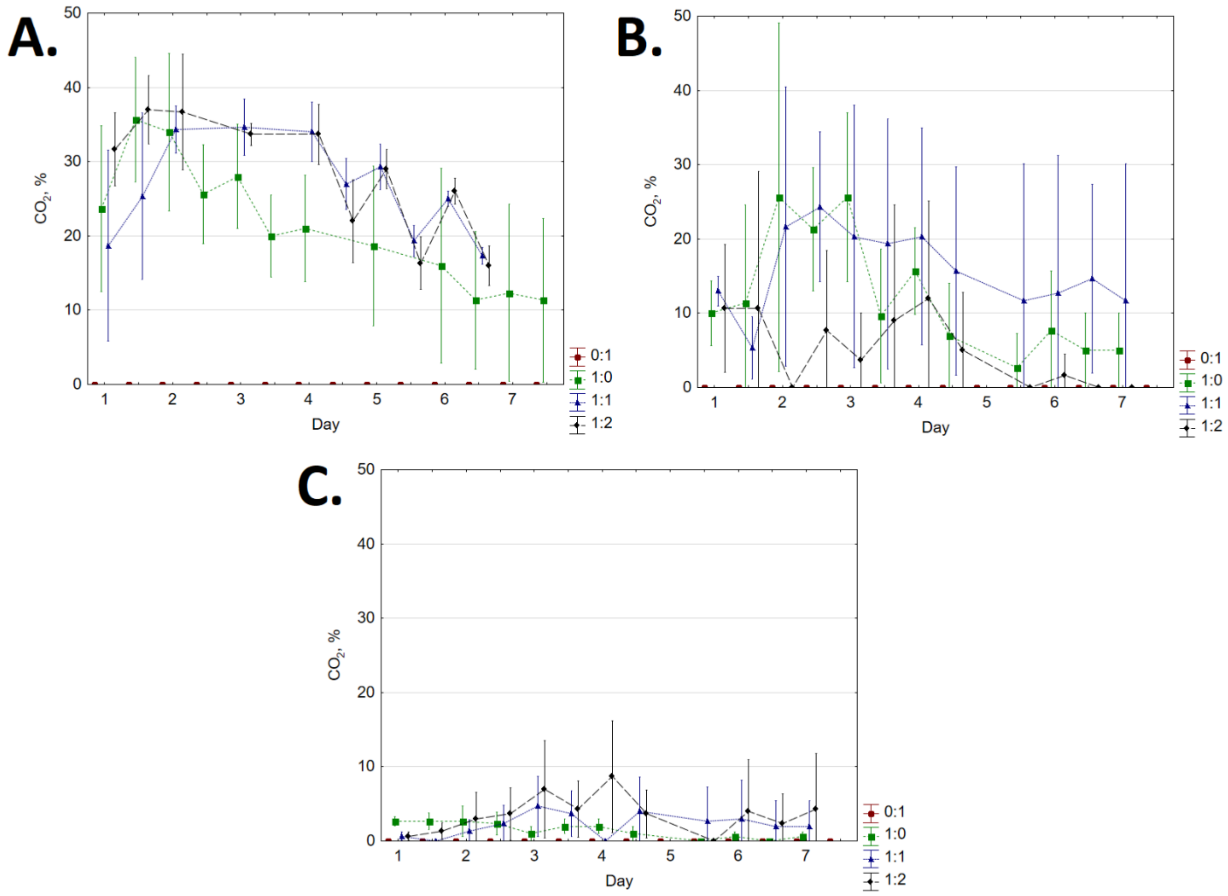

Figure S1. Carbon dioxide concentration in the bioreactors headspace during composting at: A) 45°C, B) 60°C, C) 70°C (average ± standard deviation) for four different food waste to gravelite ratios (0:1, 1:0, 1:1, 1:2)
